# Relative Susceptibility of *Vitis vinifera* Cultivars to Vector-Borne *Xylella fastidiosa* through Time

**DOI:** 10.1371/journal.pone.0055326

**Published:** 2013-02-12

**Authors:** Arash Rashed, Joyce Kwan, Breanna Baraff, Diane Ling, Matthew P. Daugherty, Nabil Killiny, Rodrigo P. P. Almeida

**Affiliations:** 1 Department of Environmental Science, Policy, and Management, University of California, Berkeley, California, United States of America; 2 Department of Entomology, University of California Riverside, Riverside, California, United States of America; 3 Citrus Research and Education Center, Department of Entomology and Nematology, University of Florida, IFAS, Lake Alfred, Florida, United States of America; University of Helsinki, Finland

## Abstract

Understanding the interactions between pathogen, crop and vector are necessary for the development of disease control practices of vector-borne pathogens. For instance, resistant plant genotypes can help constrain disease symptoms due to infections and limit pathogen spread by vectors. On the other hand, genotypes susceptible to infection may increase pathogen spread owing to their greater pathogen quantity, regardless of their symptom status. In this study, we evaluated under greenhouse conditions the relative levels of resistance (i.e. relatively lower pathogen quantity) versus tolerance (i.e. less symptom severity) of 10 commercial grapevine (*Vitis vinifera*) cultivars to Pierce’s disease etiological agent, the bacterium *Xylella fastidiosa*. Overall, no correlation was detected between pathogen quantity and disease severity, indicating the existence of among-cultivar variation in plant response to infection. Thompson Seedless and Barbera were the two most susceptible among 10 evaluated cultivars. Rubired showed the least severe disease symptoms and was categorized as one of the most resistant genotypes in this study. However, within each cultivar the degree of resistance/tolerance was not consistent across sampling dates. These cultivar and temporal differences in susceptibility to infection may have important consequences for disease epidemiology and the effectiveness of management protocols.

## Introduction

Variation in susceptibility to pathogenic infections is a function of the host genetic structure [1], however, constant genotype-by-environment interactions can influence the degree of disease expression in host plants [2]. Examples of these environmental variables include, but are not limited to, ambient incubation temperature, host nutrient status [3], and host age [4]. In natural circumstances, when variation in susceptibility exists among individuals in a given population, pathogens would effectively act as selective agents because they are expected to impact fitness of individuals differentially [5]. In agricultural systems, however, among-individual variability may be minimized by the use of a particular cultivar, where a certain genotype is desirable because of its associated economic value. In practice this can result in significant yield loss if the plant genotype is susceptible to pathogens [6], [7], [8]. However, post-infection plant resistance can be achieved through defensive mechanisms that either negatively impact the pathogen by limiting its multiplication and reducing its population (i.e. resistance), or by limiting its impact on host phenotype (i.e. tolerance). Notably, levels of resistance to infection and tolerance of infection among different host types are not necessarily correlated with each other [5], [9], [10]. The relationship between pathogen quantity (hereafter infection level) and symptom severity becomes even more crucial in vector-borne disease complexes, where the outcome of pathogen-host interactions is expected to also influence vector host choice and feeding behavior, and subsequently disease epidemiology [11].

*Xylella fastidiosa* is a vector-borne xylem-limited bacterium that infects a wide range of host plants, including several economically important agricultural crops [12], [13]. In grapevines, *X. fastidiosa* causes Pierce’s disease, which increased in prevalence in California following the establishment of an invasive leafhopper vector *Homalodisca vitripennis* (Hemiptera: Cicadellidae) [14], [15], [16], [17]. *Xylella fastidiosa* is inoculated into a healthy grapevine during insect xylem-sap feeding, then multiplies and spreads throughout the host by colonizing xylem vessels [18]. Bacterial colonization of the xylem network eventually leads to occlusion of vessels, limiting water flow through the plant [19], [20], [21], [22]. Although mechanism of pathogenicity has not been fully elucidated, water stress due to xylem blockage is an important trigger for Pierce’s disease symptoms [23]. Typically, Pierce’s disease symptoms include progressive leaf scorch, irregular cane maturation, ‘match-stick’ petioles (petiole with no leaf blade), dieback of the apex, and ultimately plant death [13], [24].

Different species of *X. fastidiosa* host plants vary in their susceptibility to infection; plum and coffee plants appear to be relatively more susceptible to infection than citrus, in that a higher proportion of their xylem vessels become colonized by the pathogen [22]. Similarly, variation in susceptibility has been reported among grape species [24], [25] with *Vitis vinifera* genotypes being more susceptible to *X. fastidiosa* infection than other *Vitis* spp. [26]. Within *V. vinifera,* different cultivars also exhibit variation in susceptibility to Pierce’s disease [24], [27], [28], [29]; this variation has been attributed to among-genotype differences in bacterial quantities and the rate of xylem occlusion along the stem tissue [20], [23], [24].

The importance of an objective quantification for the relationship between infection level and symptom severity of different *V. vinifera* cultivars became more evident in light of studies showing, *i)* the correlation between host infection level and vector transmission efficiency [30], and *ii)* vector non-random preference based on host symptoms status [31]. Vector exposure to higher bacterial quantities can increase the probability of successful pathogen acquisition, and subsequently, increase its overall transmission efficiency [30], [32]. In addition, vector preference for asymptomatic hosts [16], [31] means that tolerant genotypes - which can host high bacterial quantities and yet exhibit limited disease symptoms - may potentially function as a pathogen source for vineyards containing more susceptible cultivars (MP Daugherty, unpublished results).

In the present study the term ‘resistance’ is used as a reflection of a plant’s relative ability to limit (or reduce) infection level, whereas ‘tolerance’ reflects the extent to which a plant can maintain a healthy phenotype despite infection. Moreover, each of the above-mentioned traits is assumed to exist as a continuous metric rather than a categorical state [33]. The relative nature of the two definitions tolerance and resistance would also make between-study comparisons a difficult task, as in addition to differences between pools of evaluated cultivars, differences in experimental conditions can greatly influence bacterial population growth and symptom severity [34], [35].

This study was conducted to compare infection level and symptom severity in 10 grapevine cultivars that are commonly grown in California and in other viticultural regions across the globe. Resistance and tolerance were evaluated in each cultivar at successive time points to see whether these traits are correlated and whether their relative levels change as infection progresses.

## Materials and Methods

Two-bud cuttings of 10 grape cultivars were rooted in a mix of perlite and vermiculite (1∶1) on a misting bench in the Oxford Tract greenhouse facility at the University of California, Berkeley. This collection included 8 wine grape cultivars (cv. Barbera, Cabernet Franc, Cabernet Sauvignon, Chardonnay, Chenin Blanc, Merlot, Rubired, Zinfandel) and 2 table grape cultivars (cv. Flame Seedles, Thompson Seedless). These varieties were selected because they are among the most commonly grown cultivars in California vineyards and elsewhere. Certified pathogen free dormant cuttings of each cultivar were provided by Foundation Plant Services, University of California, Davis. Uninfected certified cuttings of different cultivars never developed disease symptom nor tested positive for *X. fastidiosa*. Following root development, cuttings were transplanted into 5-cm pots filled with Supersoil potting soil (Rod McLellan Company, San Mateo, CA, USA). Later, these rooted grapevine cuttings were transferred into 1-gallon pots filled with a mix of Supersoil (50%), sand (25%), and vermiculite (25%), and maintained in an insect free glasshouse in the same facility.

Pathogen inoculum was prepared by growing *X. fastidiosa* subspecies *fastidiosa* (STL strain) cells on PWG medium and suspending them in SCP buffer [35]. Twenty microliter of this suspension was used to mechanically needle-inoculate 6-month old grapevines at the stem base, in late December 2009. Inoculations were performed in 2 temporal blocks. Ten plants of each of the 10 cultivars were inoculated in each block. Experimental plants were regularly trimmed from the side-shoots and pruned to stay approximately 120–150 cm in height. During the experiment, temperature was set to 22–26°C. Lighting (14∶10, L:D) was supplemented by 1000 Watt, PL 2000 greenhouse lights (P.L. Light Systems Inc., Lincoln, ON, Canada). Plants were sampled every 4 weeks, starting 8 weeks post-inoculation, for 3 consecutive months. Hereafter, the period between inoculation and sampling date is referred to as ‘incubation time’. At each of the three incubation times a petiole was removed from +10-cm above the point of inoculation. In the last incubation period (week 16), samples were removed from both +10 and +90-cm above the point of inoculation. Samplings plants at the point of inoculation and also approximately 90-cm above the point of inoculation would allow detecting potential movement and new colonization of succulent vine tissue; it has been suggested that variations in susceptibility can be due to structural differences among plant cultivars, which restricts pathogen growth and movement to various degrees [23]. Plants were scored for symptoms, following a 0 to 5 scale proposed by Guilhabert and Kirkpatrick [36] (0 = asymptomatic, 1 = One or 2 leaves with scorched margins, 2 = Two to 3 more developed scorched leaves, 3 = All leaves scorched and a few match-stick petioles, 4 = All leaves heavily scorched and many match-stick petioles, 5 = A few leaves only present at the end of the cane). Symptom scores were recorded at the same time as petiole samplings in each incubation time (weeks 8, 12, and 16). Collected petioles were stored at −80°C for later bacterial quantification.

### Bacterial Quantification

Petiole samples were frozen in liquid nitrogen and ground using mortar and pestle. A robotic workstation QIAcube and a Qiagen extraction kit (Dneasy plant mini with Qiashredder, 2007), was then used to perform all DNA extractions. To maximize yield, the lysis buffer was supplemented with 0.5% lauryl sarcosine and 10% PVP-40 (polyvinylpyrrolidine). Pathogen absolute quantification within plant tissue was performed with SYBR Green Mix (Applied Biosystems) on a 7500 real-time thermocycler (Applied Biosystems) according to Daugherty *et al.* [33]; all samples were run in triplicate and results were averaged. We used primers HL5 and HL6 designed by Francis *et al.* [37]. To obtain the standard curve, *X. fastidiosa* DNA was extracted from suspensions of cultured cells while portions of these suspensions were used to plate serial dilutions to correlate the number of cells with the content of DNA. Bacterial cell numbers were estimated based on *X. fastidiosa* DNA quantity within 1 µg of total extracted DNA from petiole tissue.

### Statistical Analysis

To test for differences in symptom scores a two-way generalized linear mixed effects model (GLMM) with Poisson error distribution was used. Plant cultivar and disease incubation time were defined as fixed effects. Plant replicate identity was treated as a random effect. This model structure was needed to account for the repeated measures made on individual plants and non-normal error.

A two-way repeated-measures mixed-effects model, with diagonal repeated covariance, was used to compare infection level among grape cultivars. Cultivar and incubation time (repeated measure) were treated as fixed effect categorical variables. Tukey (Honestly Significant Difference (HSD)) was used for any *post hoc* pairwise comparisons. Inoculation block did not have a significant effect in either analysis (*P*>0.68 in both), and was therefore dropped from final models. Log-transformed infection levels provided the closest fit to the normality curve.

On week 16, infection levels of the petioles sampled at +10-cm and +90-cm from the point of inoculation were compared. Due to the lack of independence between the two-petiole samples within each plant, a two-way repeated-measures mixed-effects model with diagonal repeated covariance was also used to compare infection levels at this date. Initial model included block, grape cultivar, and sampling position (repeated measure) and cultivar-by-sampling position interaction. Block was removed from our final model, as initial analysis detected no significant effect of this variable (*F*_1, 252.2_ = 0.019; *P* = 0.890).

Pearson’s correlation (P. Corr.) was used to evaluate the relationship between infection level and symptom severity among cultivars. To do this, mean infection level was correlated with mean symptom severity for each cultivar (n = 10 cultivars). Separate correlations were conducted at each of the three time points.

Hierarchical agglomerative cluster analysis was performed to illustrate the similarity between evaluated cultivars based on their symptom severity, infection level, and the proportion of samples with zero cell counts, for each of the three incubation times, separately. This analysis describes in a relative sense the similarity among cultivar responses to infection by *X. fastidiosa* and whether cluster composition changes over the duration of infection. Calculated distances were based on Pearson correlation and clustering employed the Ward method [34], with multiscale boostrap resampling to calculate node confidence.

The mixed-effects models and correlations were performed in IBM SPSS (ver. 21).

The cluster analysis was performed using the “pvclust()” package in the R programming language. GLMM analysis was also conducted in R, using lme4 package [38].

## Results

### Infection Level and Symptom Severity

**Figure 1:**
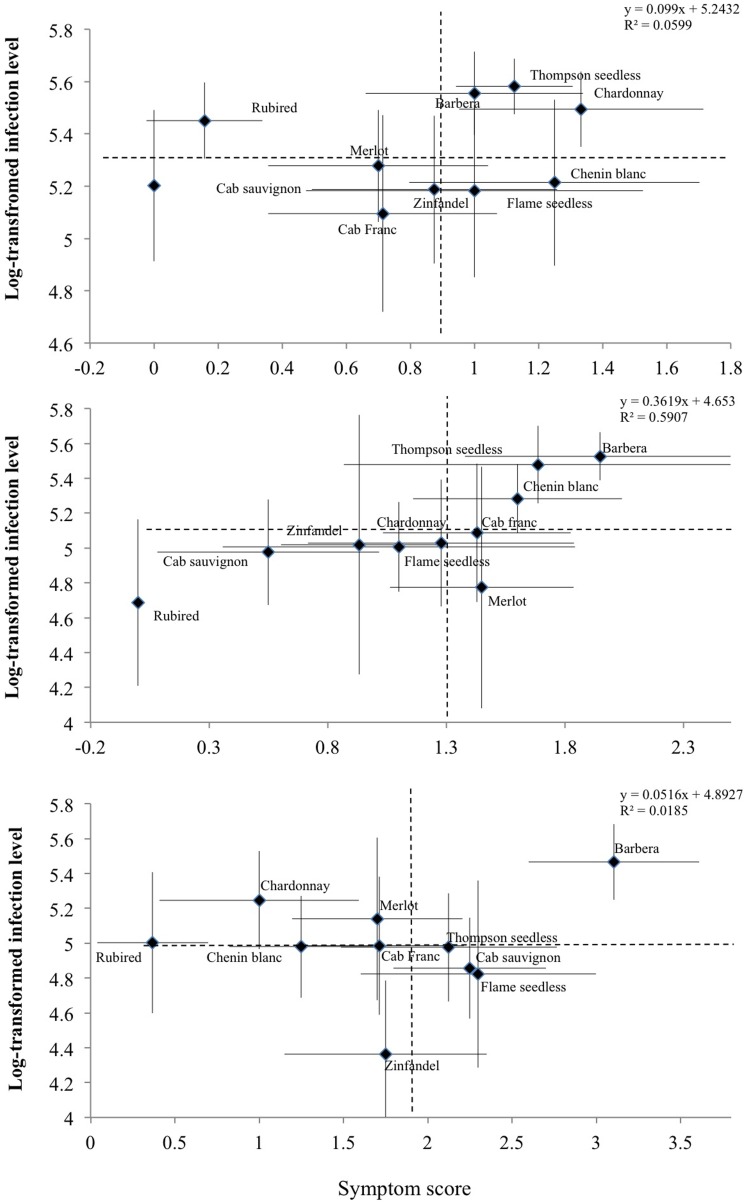
Categorization of *Vitis vinifera* cultivars in relation to their bivariate X. fastidiosa infection level and symptom severity means at A) 8, B) 12, and C) 16 weeks post inoculation. Error bars represent 95% confidence intervals. Note that the scale of axes differ among panels.

Infection level varied significantly among the 10 evaluated cultivars (*F*_9, 328.03_ = 5.48; *P*<0.001) and across three incubation times (*F*_2, 229.83_ = 16.28; *P*<0.001). Although incubation time-by-cultivar interaction was non-significant (*F*_18, 231.45_ = 1.57; *P* = 0.067), collectively, average cultivar infection levels decreased between weeks 8 and 16 (pairwise LSD, *P*<0.001), while symptom severity increased (Fig. 1). Some cultivars had consistently high infection levels (e.g., Barbera), and others showed a decrease in infection level over time (e.g., Zinfandel, Cabernet sauvignon, and Flame seedless).

**Figure 2:**
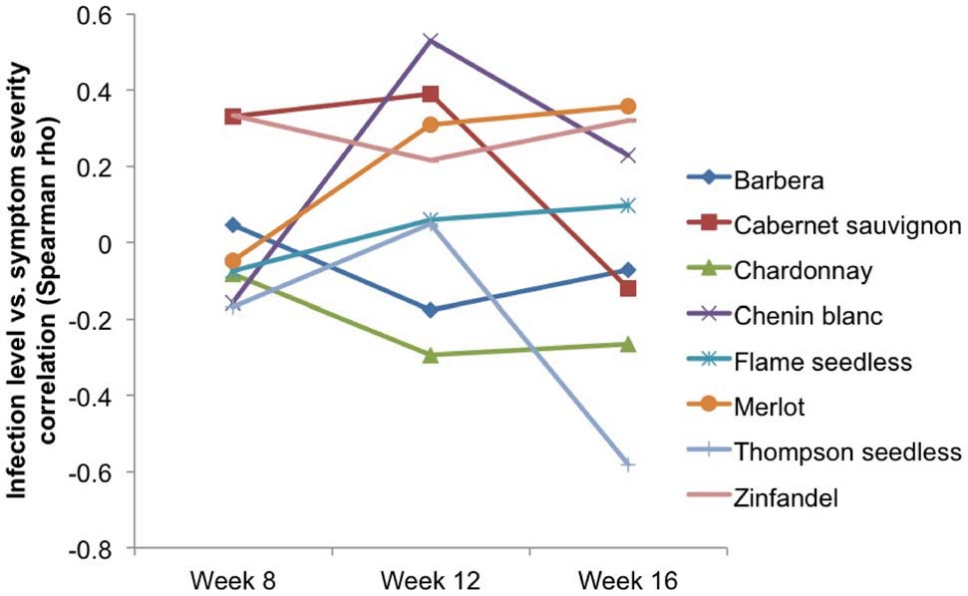
Variations in Spearman rho correlations between symptom severity and infection level of the 10 evaluated grape cultivars over time.

Results of the repeated measures GLMM showed significant differences in symptom score among cultivars (χ^2^ = 61.73, df = 9, *P*<0.0001) and incubation times (χ^2^ = 51.48, df = 1, *P*<0.0001). Yet, there were differences in symptom severity changes over time among the evaluated cultivars, as revealed by a significant cultivar-by-incubation time interaction (χ^2^ = 43.43, df = 9, *P*<0.0001; Figs 1 and 2).

In spite of a positive trend, the correlation between symptom severity and infection levels among evaluated grape cultivars on weeks 8 and 16 post-inoculation was non-significant (week 8: *P. corr.* = 0.229, *P* = 0.524; week 16: *P. Corr.* = 0.099, *P* = 0.785; Fig. 1). The relationship between symptom severity and infection level, however, was significant on week 12 (*P. Corr.* = 0.699, *P* = 0.024; Fig. 1).

Rubired exhibited modest disease symptoms, especially when compared to Barbera or Thompson seedless. Symptom severity in cultivars, such as Rubired, increased little over time whereas others, such as Barbera and Cabernet Sauvignon, increased markedly between weeks 8 and 16 (Fig. 1). In addition, the relationship between symptom severity score and infection level varied among cultivars over time (Fig. 2).

**Figure 3:**
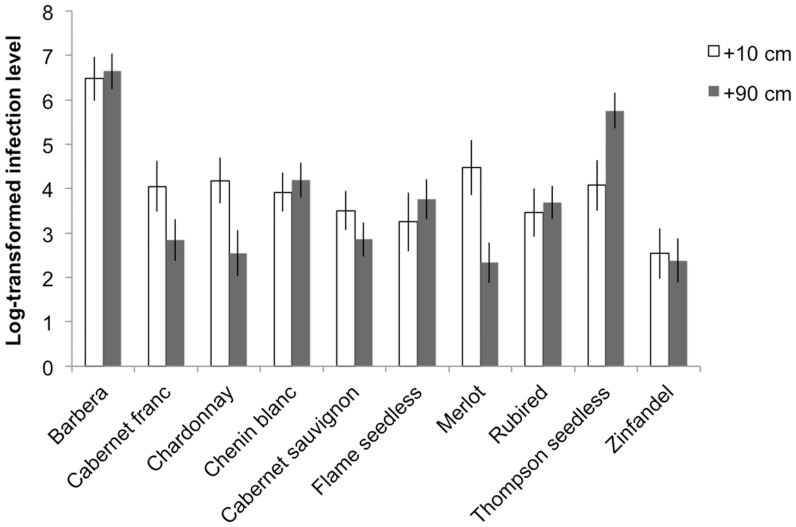
Infection levels of the petioles collected from +10 and +90 cm above the point of inoculation (POI) for each cultivar. Open bars refer to the infection levels at 10-cm above the point of inoculation. Filled bars represent the infection level of the petiole samples taken 90-cm above the point of inoculation. Error bars represent ±1 SE.

We did not detect a significant difference between infection levels at 10-cm and 90-cm from the point of inoculation among cultivars on week 16 (*F*_1, 249.5_ = 1.75, *P*<0.190; Fig. 3). However, there not only was a significant effect of cultivar (*F*_9, 248.9_ = 11.63, *P*<0.001; Fig. 3), but also variations in infection levels at the two sampling positions was cultivar dependent, as revealed by a significant sampling position-by-cultivar interaction (*F*_9, 248.9_ = 2.43, *P* = 0.012; Fig. 3). Some cultivars (e.gs., Barbera, Chenin blanc, and Rubired) showed similar levels of translocation of *X. fastidiosa* to 10 and 90-cm above the point of inoculation. Some (e.g., Thompson seedless) showed greater translocation to 90-cm, and others (e.g., Chardonnay and Merlot) had higher infection levels at 10-cm compared to 90-cm (Fig. 3).

### Relative Resistance and Tolerance of Grape Cultivars

**Figure 4:**
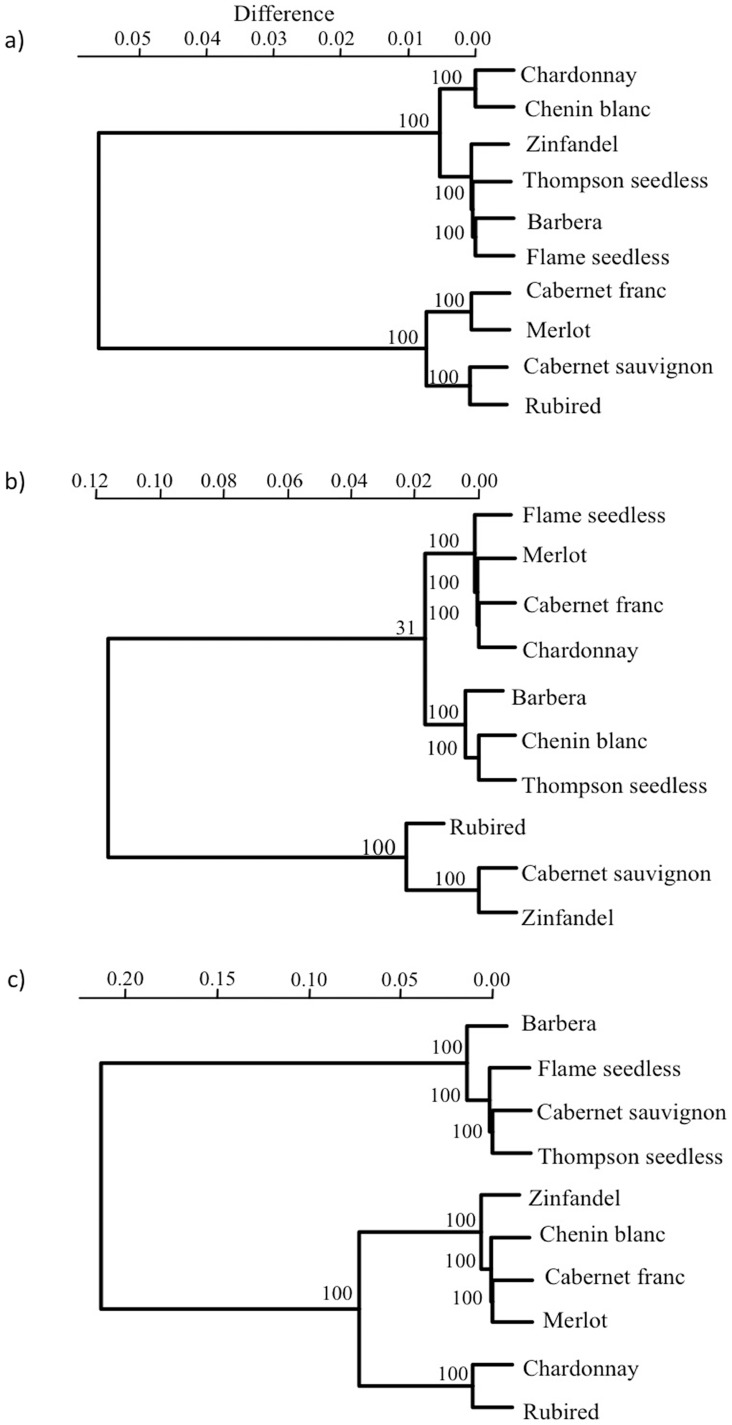
Cultivar clusters on week 8 (A), week 12 (B), and week 16 (C) showing similarity in response to *X. fastidiosa* infection. Numbers represent approximate unbiased confidence values for a given node based on multiscale bootstrap resampling.

Cultivars formed distinct groups based on symptom severity, infection level and the proportion of undetectable infections (Fig. 4). Cluster composition and the number of distinct clusters varied across the three incubation times. Cluster analysis for each sampling date revealed two well-supported distinct clusters in the first and the last incubation times (Fig. 4). In the second incubation time, three clusters were formed. The general observed pattern was that the level of resistance/tolerance was not consistent in most of the evaluated cultivars, as cluster composition changed over time (Fig. 4). Because this classification is dependent on how varieties respond to infection over time, and their relative susceptibility and tolerance in relation to each other, variation was expected unless all varieties responded equally to infection. Barbera and Thompon Seedless consistently clustered together, forming a relatively susceptible cluster that included a changing collection of other cultivars among the three time points. Conversely, Rubired was part of a relatively more resistant and tolerant cluster whose members also shifted over time. Chardonnay started off in the most susceptible cluster at week 8, was found in an intermediate cluster at week 12, and was found in the most resistant and tolerant cluster at week 16.

## Discussion

The 10 grapevine cultivars tested in this study showed variability in their susceptibility to infection as well as Pierce’s disease symptom expression. Cultivars did not show a consistent pattern of susceptibility as the correlation between symptom expression and infection level varied across sampling dates; a positive correlation between symptom score and infection level among the evaluated cultivars was observed only on week 12 post-inoculation. This finding suggests that within *V. vinifera*, the degree of cultivar resistance and tolerance varies over time.

Although for the majority of cultivars symptom severity was progressive over time, infection levels did not follow a similar pattern. For example, cultivars Flame seedless, Thompson seedless and Zinfandel showed severe symptoms on week 16, significantly more so than week 8, yet infection level in those cultivars had a negative trend across the three sampling periods. The reduction in *X. fastidiosa* infection level over time has been previously reported for several grape species [26], including *Muscadinia rotundifolia*, *Vitis girdiana*, *V. arizoniaca* and *V. nesbittiana*. However, for *V. vinifera* leaves, Fritschi and colleagues [26] observed a significant increase in the infection levels between days 34 and 77 post-inoculation followed by a non-significant increase in infection level from day 77 to 113 (estimated from Fig. 1 in reference [39]). Likewise, for Chardonnay we observed an increase in infection level between weeks 12 and 16. However, infection level for those dates was lower than that for week 8. The observed variation in bacterial populations over time indicates that among species comparisons should consider more than one cultivar per species, since within-species variability is expected.

Reductions of infection levels for some cultivars close to the point of inoculation may be due to bacterial mortality. Site-specific plant responses to infection and usage of potentially limited nutrients by cells may trigger increased mortality. Interestingly, overall bacterial quantifications of petioles sampled at 90-cm above the point of inoculation on week 16 were not statistically different from bacterial populations at the base of the shoot. Merlot, Chardonnay and Cabernet franc had higher bacterial quantities around the point of inoculation than at about 90-cm. Thompson seedless one of the most susceptible cultivars in this study, however, had a higher infection level at 90-cm above the point of inoculation than at about 10-cm, suggesting that bacterial relocation within the vascular system may determine disease severity in affected hosts as previously suggested [18], [40].

Several studies have shown that Chardonnay is a highly susceptible genotype to *X. fastidiosa* infection under both field and greenhouse conditions [20], [25], [29]. In this study, the infection level in Chardonnay followed that of Barbera and Thompson seedless, and Chardonnay was one of the most susceptible cultivars to *X. fastidiosa* infection. Chardonnay was categorized as a non-resistant/low tolerance cultivar on week 8. However, this cultivar was placed in a resistant/non-tolerant and non-resistant/tolerant category on weeks 12 and 16, respectively. In a previous greenhouse study [41], Thompson seedless and Chardonnay were also susceptible to *X. fastidiosa* infection. Flame seedless, a field-susceptible cultivar (A. H. Purcell, *personal communication*), appeared to be resistant/tolerant on weeks 8 and 12, but it moved to the resistant/non-tolerant category on week 16. The placement of this cultivar in resistant/non-tolerant category indicates that Flame seedless may have lower infection levels (bacterial populations), but is highly intolerant as it shows severe disease symptoms. Rubired had the lowest overall symptom severity and was categorized as tolerant and resistant across incubation times. Thompson seedless and Barbera were the most susceptible genotypes in the present study as they had the highest symptom score and infection levels among the rest of the cultivars. Unlike Raju and Goheen [29], who categorized Zinfandel as one of the more susceptible and Chenin Blanc as the least susceptible cultivar among 25 tested genotypes, Zinfandel was one of the least susceptible cultivars along with Rubired, and it showed both low infection levels and low symptom severity across the three sampling dates. Chenin Blanc, on the other hand, was categorized as highly intolerant on week 8 and nonresistant/intolerant on week 12. Chenin Blanc showed average levels of resistance and higher levels of tolerance on week 16 post-inoculation. Establishing a classification of resistance/tolerance for *V. vinifera* cultivars is challenging as both terms are relative and depend on the subset of cultivars that are being evaluated in any given study. Thus, including a greater number of cultivars in individual comparative studies provides the opportunity for comparing relative susceptibility in a defined condition, especially within-species, where among-genotype differences may be more difficult to detect.

It has been shown that bacterial population growth is influenced by environmental conditions [34]. Among-study differences in experimental conditions may, at least partially, explain the observed inconsistencies. Moreover, both susceptibility and tolerance are relative terms and direct between-study comparisons may vary depending on the pool of cultivars that are being evaluated. Studies on susceptibility and tolerance of grapevines to Pierce’s disease should not be generalized to all viticultural localities. Instead, independent field studies must be conducted in the geographical location of interest, with the set of available and/or preferred cultivars. Lastly, reductions in bacterial population may be a consequence of physiological changes in plants triggered by environmental conditions. Although experiments were performed in the greenhouse with sufficient temperature and lighting, we believe that this may be a possibility.

Plant samplings were consistently conducted at the point of inoculation proximity throughout the study and across sampling dates. Moreover, bacterial quantity comparison at the final sampling date revealed no significant variation between petioles collected from two different plant sites. However, it is known that *X. fastidiosa* is heterogeneously distributed within infected grapevines [42], and thus our quantification of infection levels based on a single petiole may not be a precise representation of infection levels. Although petioles are known to harbor higher bacterial quantities than stem [24], infection level of the stem tissue may better reflect among-cultivar variations [24], [25]. Nonetheless our quantitative PCR approach using petiole tissue, consistently sampled at the point of inoculation, was sensitive enough to detect variations among *V. vinifera* genotypes and across sampling dates.

The lack of correlation between symptom severity and infection level among cultivars indicates the existence of phenotypic diversity within *V. vinifera* in relation to *X. fastidiosa* infection. In addition to its role in inferring direct damage inflicted by Pierce’s disease, evaluating tolerance and resistance of commonly used cultivars in a specific region is of significant importance from the epidemiological point of view [11], [43]. For example, tolerant cultivars that harbor high bacterial populations may result in significant pathogen spread, eventually leading to high disease incidence. In other words, highly resistant cultivars should contain pathogen spread, but highly tolerant ones may promote it (MP Daugherty, unpublished results).

In conclusion, the relationship between symptom severity and bacterial population varied through time, and among-study variations indicate that environmental variables could determine the outcome of *X. fastidiosa*-by-grape genotype interactions. Changes in plant attractiveness [44] and nutritional quality [45] in response to infection can affect vector host choice behavior and consequently influence pattern of pathogen distribution. A recent study showed that sharpshooters discriminate against symptomatic grapevines but that their host choice is not influenced by the presence of *X. fastidiosa* [31]. Additionally, bacterial populations within the infected host directly affect vectors’ exposure to the pathogen and thus can increase transmission rate by the vector [30], [32]. The observed variability in symptom development among grapevine genotypes and across incubation times indicates the importance of phenological studies among vectors, host genotypes, and bacterial populations within source plants.
